# Role of Deubiquitinases in Human Cancers: Potential Targeted Therapy

**DOI:** 10.3390/ijms21072548

**Published:** 2020-04-06

**Authors:** Keng Po Lai, Jian Chen, William Ka Fai Tse

**Affiliations:** 1 Guangxi Key Laboratory of Tumor Immunology and Microenvironmental Regulation, Guilin Medical University, Guilin 541004, China; kengplai@cityu.edu.hk; 2 Center for Promotion of International Education and Research, Faculty of Agriculture, Kyushu University, Fukuoka 819-0395, Japan

**Keywords:** deubiquitinase, degradation, therapeutic target, cancer

## Abstract

Deubiquitinases (DUBs) are involved in various cellular functions. They deconjugate ubiquitin (UBQ) from ubiquitylated substrates to regulate their activity and stability. Studies on the roles of deubiquitylation have been conducted in various cancers to identify the carcinogenic roles of DUBs. In this review, we evaluate the biological roles of DUBs in cancer, including proliferation, cell cycle control, apoptosis, the DNA damage response, tumor suppression, oncogenesis, and metastasis. This review mainly focuses on the regulation of different downstream effectors and pathways via biochemical regulation and posttranslational modifications. We summarize the relationship between DUBs and human cancers and discuss the potential of DUBs as therapeutic targets for cancer treatment. This review also provides basic knowledge of DUBs in the development of cancers and highlights the importance of DUBs in cancer biology.

## 1. Introduction

Deubiquitinases (DUBs) deconjugate ubiquitin (UBQ) from ubiquitylated substrates to regulate their activities and stability. They are a heterogeneous group of cysteine proteases and metalloproteases [1] that cleave the isopeptide bond between a lysine and the C-terminus of UBQ. DUBs can also edit UBQ chains and process UBQ precursors. In addition, some DUBs can edit UBQ-like proteins and their conjugates. DUBs in the human genome can be classified into subclasses based on their UBQ-protease domains [1]: UBQ-specific proteases (USPs), which represent the largest class, otubain proteases (OTUs), UBQ C-terminal hydrolases (UCHs), Machado–Joseph disease proteases (MJDs), Jab1/Mov34/Mpr1 Pad1 N-terminal+ (MPN+) (JAMM) motif proteases, and motif interacting with ubiquitin-containing novel DUB family (MINDY) [2]. In addition, some new potential DUBs without the above typical domains were currently identified, such as the monocyte chemotactic protein-induced protein (MCPIP) [3] and Zn-finger and UFSP domain protein (ZUFSP) [4]. Approximately 100 DUBs have been identified in humans. They are expressed and located in various organelles in the cell [5]: USP1 and USP7 are found in the nucleus, USP30 in the mitochondria, and USP21 and USP33 in microtubules. More examples are shown in Table 1 [5,6,7,8]. Some DUBs have higher expressions in specific tissues, such as USP3 and UCHL3 in the pancreas and lung and USP14 in the brain [5]. 

There has been extensive research on ubiquitination [9,10] and how DUBs regulate the deubiquitylation process and their relative functions [11]. Moreover, an increasing number of studies have uncovered the role of DUBs in cancer development [12]. Numerous informative reviews on DUBs have been published [13,14,15,16,17,18] and research on DUBs has been increasing in recent years. In this review, we aim to provide enriched content that summarizes the classical discoveries, and includes the current findings on DUBs that are related to different aspects of human cancer, including proliferation, cell cycle control, apoptosis, the DNA damage response (DDR), tumor suppression, oncogenesis, and metastasis. Summarized information is shown in Table 2. Lastly, we discuss the potential of DUBs as chemotherapeutic targets for cancer treatment.

## 2. DUBs and Cell Cycle Control

The cell cycle refers to a series of processes, including DNA synthesis, S phase; cell growth, G1 phase; evaluation of the accuracy of the genomic material, G2 phase; and cell division, M phase. The cycle is completed by duplicating the genetic information and equally segregating it into two daughter cells. Many cell cycle checkpoints are controlled by cyclins and cyclin-dependent kinases (CDKs) [19]. The E3 ligases participate at almost every phase, indicating the importance of ubiquitination and deubiquitination in regulating the cell cycle [20,21].

**Figure 1:**
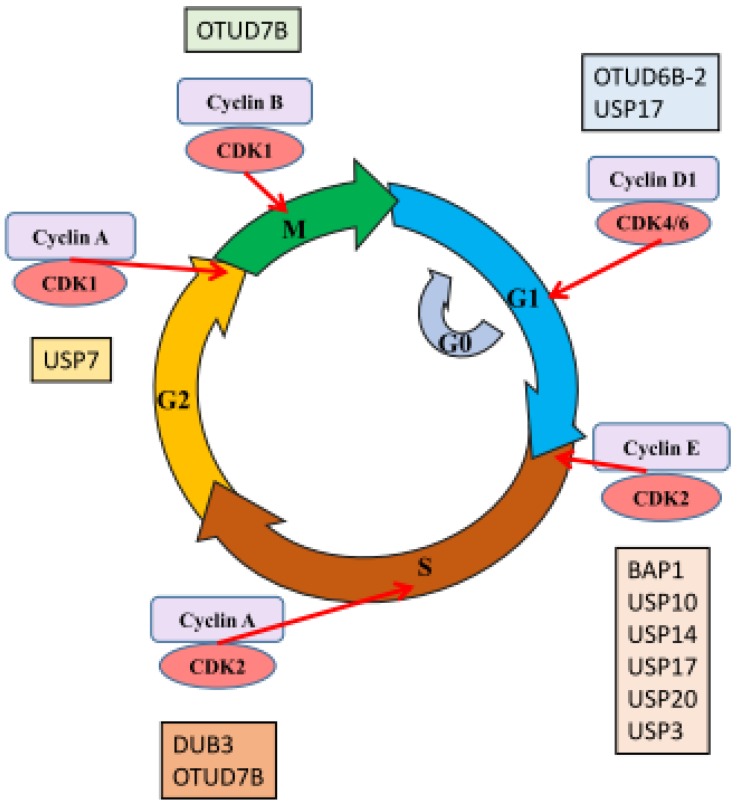
Roles of DUBs in cell cycle control in cancers. The eukaryotic cell cycle consists of the G1 phase (blue), the S-phase (brown), the G2 phase (yellow), and the M (mitosis) phase (green). Cells can enter a quiescent state, the G0 phase (grey). Cell cycle phases are indicated by different colored arrows. The cell cycle is regulated by complexes that are composed of cyclins (light purple), and its relative cyclin-dependent protein kinases (CDKs) (pink). The cyclin-CDK complex plays regulatory roles in the cell cycle. The red arrows indicate their targets, either within the designated cell cycle phase or in the transition state. Various DUBs have been shown to interact with the cyclin–cdk complex. DUBs that participate in G1 phase are labeled in light blue; S phase in light brown; G2 phase in light yellow; and M phase in light green. The detailed interaction partner of each individual DUB can be found in the main text and the Table 2.

The ability to advance through different stages of the cell cycle regardless of inhibitory signals is one of the hallmarks of cancer. A large number of DUBs have been found to play roles in cell cycle control of cancers via the regulation of different cell cycle checkpoints. OTUD6B-2 and USP17 were reported to control the G1 phase; USP3, USP10, USP14, USP17, USP20, and BAP1 played roles in the G1/S transition. In addition, S/G2 transition was controlled by OTUD7B and DUB3. USP7 and OTUD7B were necessary for the regulation of mitotic phase (Figure 1). 

For the G1 phase regulation, OTUD6B operates downstream of mTORC1 signaling in non-small cell lung cancer (NSCLC), and its isoform OTUD6B-2, was reported to control the stability of cyclin D1 and c-Myc [24]. USP17 is another cell cycle-regulating DUB. It was found to be highly expressed in colon, esophageal, and cervical cancers. The depletion of USP17 increases the levels of the CDK inhibitor p21 and impairs the G1-S transition, leading to cell cycle arrest [31]. In addition, USP17 deubiquitinates the transcription factor ELK-1. The stabilization of ELK-1 increases the expression of cyclin D1 [32]. USP17 further decreases Su(var)3-9, enhancer-of-zeste, and trithorax domain-containing protein 8 ubiquitination to trigger cellular senescence [33].

For the G1/S phase, USP20 deubiquitinates and stabilizes the DNA checkpoint protein claspin, and thus activates the ATR-Chk1 signaling in the DNA damage response pathway [98]. USP10 deubiquitinates SKP2 and augments the activation of Bcr-Abl by mediating deubiquitination and stabilization of SKP2 in chronic myelogenous leukemia cells [29]. An RNAi-based screening study discovered that USP21 binds and deubiquitinates FOXM1, leading to its increased stability, which induces cell cycle progression in basal-like breast cancer [34]. In addition, DUBs could regulate transcription factors for cell cycle control. The transcription factor Krüppel-like factor 5 (KLF5), which promotes cell proliferation by inhibiting the expression of the cell cycle inhibitor p27 [22], is highly expressed in breast cancer. A genome-wide siRNA library screen identified BAP1 and USP3 as KLF5 DUBs. Both BAP1 and USP3 bind to and stabilize KLF5 via deubiquitination [22,35], indicating the possible regulatory role of DUBs in cancer proliferation. Another example is the androgen receptor (AR), a key transcription factor in the development of breast cancer [99]. It has been reported that AR can be stabilized by USP14, and depletion of USP14 reduces cell proliferation by blocking the G0/G1–S phase transition in AR-responsive breast cancer cells [30]. 

For the S/G2/M phase, OTUD7B, also called cezanne, is frequently overexpressed in different cancer types, such as breast and lung cancer [100,101]. It is reported to be a cell cycle-dependent DUB because it deubiquitylates substrates of the mitotic cyclin anaphase-promoting complex/cyclosome (APC/C) and prevents their degradation during mitosis [25]. The APC/C is a key regulator of cell cycle progression through the regulation of CDK activity [26]. OTUD7B controls the cell cycle through HIF2α and E2F1 in response to oncogenic signaling [27]. In addition, it removes UBQ from GβL in the mTOR complex to regulate mTORC2 signaling in response to growth signals [28]. Besides, DUB3 can directly deubiquitinate cyclin A in NSCLC. The depletion of DUB3 decreases cyclin A levels, leading to cell cycle arrest at the G0/G1-S phase checkpoint in NSCLC cells [23]. Lastly, it is known that histone demethylases can regulate the cell cycle through transcriptional regulation [102]. The histone demethylase PHF8 is stabilized by USP7, leading to the upregulation of cyclin A2, which is critical for cell growth and proliferation in breast carcinomas [36].

## 3. DUBs and Cell Proliferation

In addition to their role in regulating the cell cycle, DUBs have been reported to regulate cell proliferation through different cell signaling pathways, such as Wnt/β-catenin signaling, p53-mouse double minute 2 (MDM2) signaling, PI3K-Akt signaling, AR signaling, and transforming growth factor beta (TGF-β) signaling. Aberrant canonical Wnt/β-catenin signaling is tightly associated with many solid and liquid tumors [103]. Furthermore, alteration or loss of differentiation control could facilitate the development of metastatic traits during tumorigenesis [104,105]. Numerous studies have demonstrated the control of Wnt/β-catenin signaling by DUBs in cancer [48,106]. USP6NL is elevated in colorectal cancer (CRC) and regulates β-catenin accumulation. Knockdown of USP6NL results in inhibition of cell proliferation and G0/G1 cell cycle arrest in human CRC cell lines [53]. In addition, USP4 is a candidate for a β-catenin-specific DUB. There is a positive correlation between the levels of USP4 and β-catenin in human colon cancer tissues. Further, knockdown of USP4 reduces invasiveness and migration in colon cancer cells [48]. β-catenin is also stabilized by USP9X, leading to high-grade glioma cell growth. USP9X removes the Lys48-linked polyubiquitin chains from β-catenin to prevent its proteasomal degradation. Depletion of USP9X induces G1-S cell cycle arrest and inhibits cell proliferation in glioblastoma cells [59].

**Figure 2:**
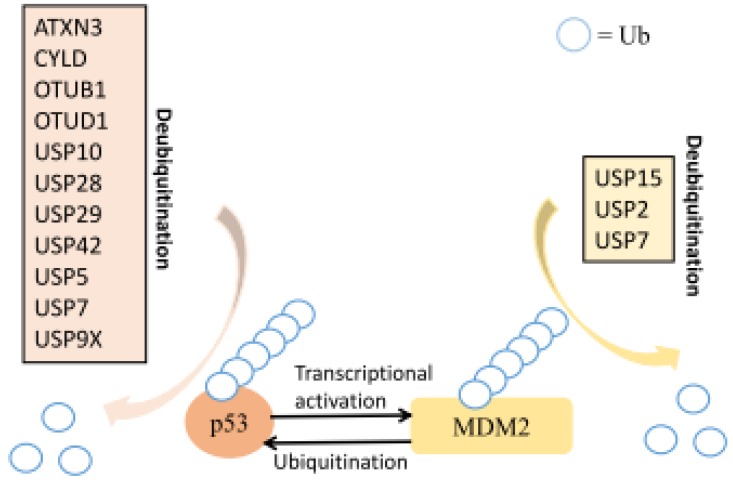
DUBs in MDM2-p53 pathways. Ubiquitination is found on both p53 and MDM2 molecules; various DUBs could revise that via deubiquitination to regulate the p53 pathway. DUBs’ targets on p53 are labeled in light brown; those that interact with MDM2 are labeled in light yellow. Detailed descriptions can be referred to the main text.

The tumor suppressor p53 is a transcription factor able to control important cellular pathways. It prevents genome mutation and plays protective roles in tumor onset and progression. It is mainly regulated by ubiquitylation, indicating the importance of DUBs in monitoring its ubiquitin cycle [107]. Both MDM2 and p53 are targeted by different DUBs (Figure 2). Suppression of USP2 leads to MDM2 destabilization and results in p53 activation [44]. USP7 plays a key role in the p53 pathway by stabilizing both MDM2 and p53 (Figure 2) [54,55,56,57,58]. Under normal conditions, USP7 has a higher binding affinity to MDM2, the major E3 ligase of p53 [56], and thus deubiquitylates MDM2 more efficiently to prevent its self-degradation and maintain stable protein levels for controlling p53 via the UBQ-proteasome pathway [108]. USP10 regulates p53 localization and stability by deubiquitinating p53. It reverses MDM2-induced p53 nuclear export and degradation [40]. Moreover, USP29 is reported to cleave poly-ubiqutin chains from p53 and thus stabilize it [47], while USP15 stabilizes the E3 UBQ ligase MDM2 in cancer cells and regulates p53 function and cancer cell survival. Inhibition of USP15 induces apoptosis and boosts antitumor T cell responses in tumor cells [42]. Furthermore, a large number of DUBs have been found to target p53 or p53-associated proteins directly, leading to proliferation. USP5 regulates p53 levels and alters cell growth and cell cycle distribution associated with p21 induction in melanoma cells [52]. OTUD1 is required for p53 stabilization, and OTUD1 overexpression increases the cleavage of caspase-3 and PARP and subsequently increases apoptosis [38]. Another p53-associated DUB, otubain 1 (OTUB1), is expressed in high-grade tumor types, such as lung, breast, and ovarian tumors. OTUB1 regulates p53 to promote tumor cell survival and proliferation [37]. USP42 controls the level of p53 ubiquitination during the early phase of the DDR to promote DNA repair, resulting in the activation of p53-dependent transcription and cell-cycle arrest in response to stress [45]. In addition, USP28 depletion leads to increased ubiquitinated H2A-K119 and decreased expression of p53, p21, and p16INK4a, suggesting a role for USP28 in cell proliferation via the control of p53 and p53-associated proteins [109]. Additionally, USP28 deubiquitinates TP53-binding protein 1 to promote p53-mediated transcription [46]. USP4 is a potential oncogene that inhibits p53 and NF-κB via histone deacetylases 2 (HDAC2) [49,50]. USP9X-dependent p53 degradation was observed in hepatocellular carcinoma (HCC) cells treated with the small molecule DUB inhibitor WP1130 [60].

DUBs are also involved in other signaling pathways that promote tumor proliferation. USP15, which stabilizes the type I TGF-β receptor and enhances the TGF-β pathway, is upregulated in various cancers [43]. In addition, OTUD1 mitigates TGF-β-induced pro-oncogenic responses via deubiquitination of SMAD7 at lysine 220 in breast cancer [39]. USP49 regulates the Akt pathway through the stabilization of FKBP51. FKBP51 activates PH domain leucine-rich-repeats protein phosphatase (PHLPP) to dephosphorylate Akt, which inhibits pancreatic cancer cell proliferation [51]. The AR pathway is commonly activated in prostate cancer (PCa), and it plays a critical role in PCa growth and progression. USP14 was reported to bind with and stabilize AR in androgen-responsive PCa cells. Overexpression of USP14 promotes the proliferation of LNCaP cells [41]. Furthermore, DUBs control different growth factors in tumor cells. For instance, USP8 prevents degradation of the epidermal growth factor receptor and thus promotes proliferation [110]. 

## 4. DUBs and Apoptosis

The ability to evade apoptosis is one of the essential changes in cancer cells that causes malignant transformation [111]. Apoptosis is a cellular self-destruction program in response to various cellular stresses. The two extrinsic and intrinsic pathways in apoptosis both involve the activation of caspase molecules. The activation of initiator caspase will further lead to the activation of executioner caspase in apoptosis [112]. DUBs were found to target different pro- and anti-apoptotic proteins in both the extrinsic and intrinsic pathways. ATXN3 stabilizes p53 by deubiquitination and promotes p53-mediated apoptosis [61]. USP5 targets p53-unanchored UBQ polymers and regulates p53-mediated transcription. Depletion of USP5 controls tumor necrosis factor alpha apoptosis-inducing ligand (TRAIL)-mediated apoptotic responsiveness in TRAIL-resistant tumor cells, and this function of USP5 ubiquitination can be blocked by caspase 8-specific inhibitors [63]. In addition, USP5 deubiquitinates the MAF bZIP transcription factor and prevents its degradation. Knockdown of USP5 leads to apoptosis in multiple myeloma cells [64]. In a chemoresistant xenograft model, JOSD1 was identified to be upregulated during the development of chemoresistance. Moreover, JOSD1 has been reported to deubiquitinate and stabilize MCL1, which plays a suppressive role in mitochondrial apoptotic signaling. Therefore, depletion of JOSD1 leads to severe apoptosis in gynecological cancer cells through the degradation of MCL1 [62]. There are several DUBs that regulate the apoptotic pathways via BCL-2 family, an inhibitor of apoptotic proteins (IAPs) and caspases. DUB3/USP17 induces apoptosis through caspase 3 activation [113], whereas USP15 plays a role in stabilizing procaspse 3 [114]. Besides, A20, a DUB belongs to the OTU subclass, interacts with caspase 8 to reverse the ubiquitination of a cullin 3-based E3 ligase [115]. As for the IAPs, they are a class of proteins that inhibit apoptosis. They contain the baculovirus IAP repeat domain and the RING domain that provides the E3 ligase property [116]. USP19 stabilizes the cellular IAP1 and cellular IAP2 during caspase activation and apoptosis [117]; OTUD1 was found to regulate the TNF-dependent cell death by modulating the cellular IAP1 stability [118]. Furthermore, USP9X was reported to interact with an E3 ligase X-linked IAP for mitotic cell fate decision [119]. In addition, USP27X was found to interact with the BIM. BIM is a pro-apoptotic BH3-only protein that regulates the cell death proteins such as BAX. Overexpression of Usp27x reduces BIM ubiquitination, and induces apoptosis in tumor cells. On the other hand, suppression of USP27X could reduce apoptosis [120].

## 5. DUBs and the DDR

**Figure 3:**
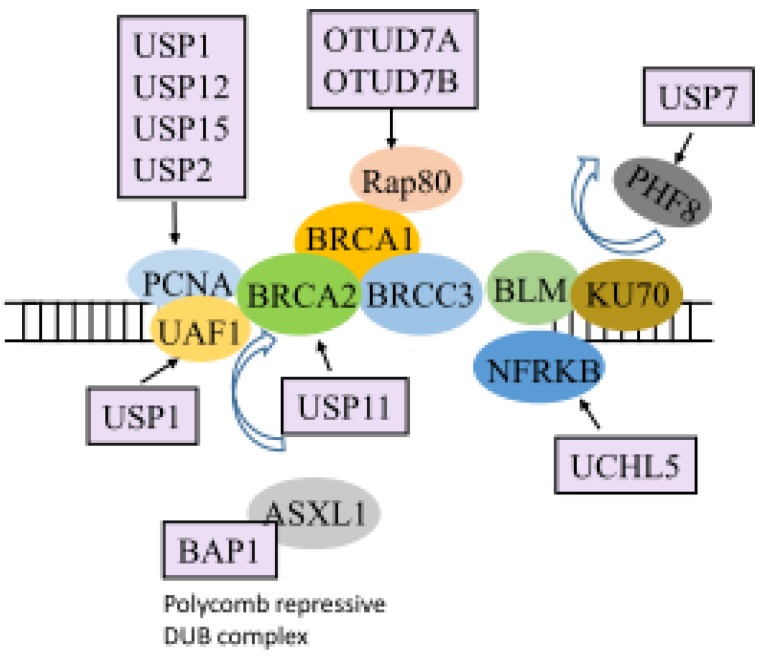
Roles of DUBs in DNA damage response. Various DUBs (light purple) have been shown to interact with molecules (various colors) that play roles in DNA repair and chromosomal stability during DNA damage. Proliferating cell nuclear antigen (PCNA) plays important roles during DNA replication and repair, while BRCA members are the key players in repairing the DNA lesions such as DNA double-strand breaks. In addition, BLM repairs DNA double-strand breaks to maintain genome stability. Detailed information can be found in the main text.

Cells undergo DDR to sense and repair unique lesion structures in the damaged DNA. Efficient DDR protects cells from genomic instability [121,122]. Ubiquitination regulates DDR by controlling DDR protein localization, activity, and stability [123]. DUBs play critical roles in different stages of the DDR through the regulation of many molecules involved in DNA repair (Figure 3). DNA repair is important for preventing tumor formation [124]. Proliferating cell nuclear antigen (PCNA) is a key molecule that mediates the tolerance to DNA damage and allows the growth of tumors. PCNA is monoubiquitinated in response to DNA damage. A fission yeast study showed the importance of UBP2, UBP12, and UBP15 in the stabilization of mono, di, and polyubiquitylated forms of PCNA, which sensitize cells to DNA damage [69]. In addition, PCNA can be deubiquitinated by USP1 in the crosslink repair pathway in Fanconi anemia [71,72,73]. In a complex with its cofactor UAF1, USP1 reverses PCNA ubiquitination [74]. UCHL5 regulates double-strand break (DSB) resection and repair by homologous recombination through protecting its interactor, NFRKB, from degradation [70]. In addition, USP20 plays role in genome maintenance and DNA repair by enhancing recombinational repair of collapsed replication forks [125]. Furthermore, USP9X regulates the DNA checkpoint protein claspin during S phase, suggesting a role in DNA repair [79]. USP7-promoted PHF8 stabilization confers cellular resistance to genotoxic insults and is required for the recruitment of BLM and KU70, which are both essential for DNA DSB repair [36].

Breast-cancer susceptibility gene (BRCA) 1 contributes to DNA repair and the maintenance of chromosomal stability in response to DNA damage [126]. BRCA1 appears to play roles in two distinct pathways of DSB repair, non-homologous end joining and homology-directed repair, through the regulation of different effectors. It has been reported that several DUBs can regulate BRCA1. The BRCA1-associated DUB BAP1 is mutated in mesothelioma and melanoma [65]. BAP1 is a phosphorylation target for the DDR kinase ATM, and BAP1 mediates rapid poly(ADP-ribose)-dependent recruitment of the polycomb DUB complex PR-DUB to repair DNA DSBs [65]. In addition, both cezanne (OTUD7B) and cezanne2 (OTUD7A) promote the recruitment of the Rap80/BRCA1-A complex by binding to Lys63-polyubiquitin and targeting Lys11-polyubiquitin in response to DNA repair [68]. Another DUB, USP11, forms a complex with BRCA2. It deubiquitylates the partner and localizer of BRCA2 to enhance DNA repair [75]. BRCA1/BRCA2-containing complex 3 (BRCC3) is a Lys63-specific DUB involved in the DDR. BRCC3 inactivation increases the release of several cytokines, including G-CSF, which enhances proliferation in AML cell lines [127]. Further, OTUD5, a specific stabilizer of the UBR5 E3 ligase, is reported to localize at DNA DSBs. OTUD5 plays two roles at DSBs. First, OTUD5 interacts with UBR5 and represses RNA Pol II-mediated elongation and RNA synthesis. In addition, OTUD5 interacts with the FACT component SPT16 and antagonizes histone H2A deposition at DSBs [67]. 

Histone ubiquitination at DNA breaks is required for activation of the DDR and DNA repair. BRCA1-BARD1-catalyzed ubiquitination of histone H2A primes chromatin for repair by homologous recombination during the DDR. Ubiquitination of histone H2A and γH2AX by the UBQ ligases RNF168 and RNF8 generates a cascade of ubiquitination. USP3 deubiquitinates ubiquitinated γH2AX and H2A [76]. USP48 is another H2A DUB that is specific for the C-terminal BRCA1 ubiquitination site. USP48 promotes genomic stability by antagonizing the BRCA1 E3 ligase function. Depletion of USP48 increases the distance between p53-binding protein 1 (53BP1) from the DNA break point [77]. It should be noted that histone ubiquitination by RNF168 is a critical event for the recruitment of BRCA1 and 53BP1, and the stability of RNF168 can be regulated by USP7. Depletion of USP7 impairs H2A and γH2AX monoubiquitination, leading to decreases in the levels of pBmi1, Bmi1, RNF168, and BRCA1 under ultraviolet radiation-induced DNA damage [78]. Moreover, USP3, a histone H2A DUB, negatively regulates UBQ-dependent DDR signaling through regulation of chromatin ubiquitination in response to genotoxic stress [128]. Lastly, CYLD deubiquitinates p53 and facilitates its stabilization in response to genotoxic stress. Loss of CYLD catalytic activity causes impaired DNA damage-induced p53 stabilization and activation of skin tumorigenesis [66]. 

## 6. DUBs and Tumor Suppressors/Oncogenes

DUBs play an important role in cancer development by controlling various different tumor suppressors and oncogenes. CYLD was first identified as the tumor suppressor gene for cylindromatosis [129]. Its protein expression level is downregulated in various tumor types [130,131]. CYLD plays an essential role in NF-κB [82] and c-Jun N-terminal kinase pathways [132]. Briefly, it inhibits NF-κB activation by promoting deubiquitylation of several UBQ-dependent NF-κB positive regulators, such as tumor necrosis factor receptor-associated factor 2 and the NF-κB essential modulator/IKKγ subunit [80,81,82]. Enhanced and/or prolonged NF-κB signaling due to reduced CYLD activity increases cellular apoptosis resistance and the chances of tumor formation [133]. USP13 also acts as a tumor suppressor through its regulation of the phosphatase and tensin homolog deleted on chromosome 10 (PTEN)/AKT pathway in oral squamous cell carcinoma. Overexpression of USP13 induces PTEN expression and represses the activation of AKT, glucose transporter-1, and hexokinase-2, leading to growth inhibition [84]. In an RNAi screen, USP11 was identified as a promyelocytic leukemia (PML) regulator to deubiquitinate and stabilize PML, counteracting the functions of PML. UBQ ligases RNF4 and the KLHL20-Cullin 3-Roc1 complex [83]. This complex causes suppression of PML in many cancer types [83]. PHLPP is a family of Ser/Thr protein phosphatases that serve as tumor suppressors by negatively regulating AKT. In CRC, USP46 is reported to bind to PHLPP and directly remove its polyubiquitin chains, resulting in the stabilization of PHLPP. USP46-mediated stabilization of PHLPP subsequently inhibits AKT, blocking proliferation and tumorigenesis in colon cancer cells [85].

A large number of DUBs have been reported to bind with and stabilize oncogenes, such as c-MYC. USP22 promotes deubiquitination of c-MYC in breast cancer cells, resulting in increased levels of c-MYC. Overexpression of USP22 stimulates tumorigenic activity in breast cancer cells and is closely correlated with breast cancer progression [87]. USP9X acts as an FBW7 interactor, and the loss of FBW7 has been observed in many types of human cancer [134]. USP9X antagonizes FBW7-mediated ubiquitylation and causes FBW7 stabilization. USP9X suppresses tumor formation by regulating FBW7 protein stability, which reduces c-MYC levels [89]. The degradation of the oncogene product MYC is also enhanced by USP28 [88]. An integrated genomic analysis of malignant pleural mesotheliomas uncovered somatic inactivating mutations in the tumor-suppressive nuclear DUB BAP1. BAP1 targets histones with the polycomb repressor subunit ASXL1 [86].

## 7. DUBs and Metastasis

**Figure 4:**
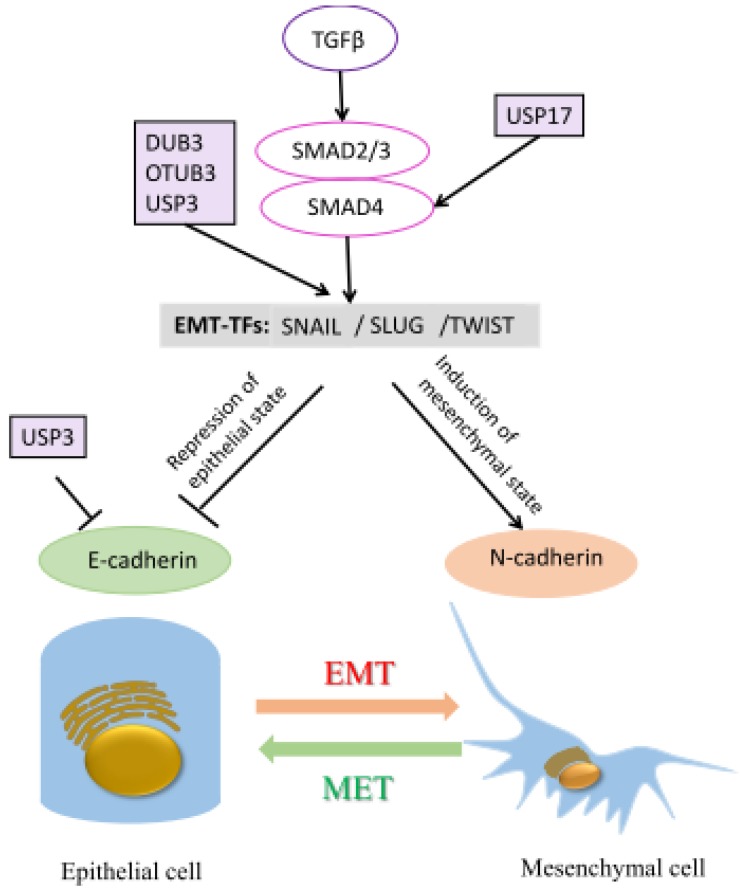
Roles of DUBs in epithelial–mesenchymal transition (EMT) in cancer metastasis. Epithelial cells are held together by numerous proteins, including tight junctions, adherens junctions, and desmosomes. These cells express molecules that are associated with the epithelial state, such as E-cadherin in epithelial state, and N-cadherin in mesenchymal state. Induction of EMT induces different EMT-inducing transcription factors (EMT-TFs) such as SNAIL, SLUG, and TWIST. These factors can then inhibit the epithelial state-related genes, such as E-cadherin, and activate the mesenchymal state related genes, such as N-cadherin. Various DUBs have been shown to interact with different EMT regulators. EMT is a reversible process, and mesenchymal cells can revert to the epithelial state by undergoing mesenchymal–epithelial transition (MET). A detailed description can be found in the main text.

Metastasis, which is the ability of cancer cells spread to different tissues or organs, is regulated by many mechanisms. It is a series of biological processes including various invasion-metastasis cascades. Multiple reports have suggested the role of DUBs in controlling these mechanisms. The epithelial–mesenchymal transition (EMT) represents one of the most important invasive events in cancer metastasis. It refers to a change of a subset of adhesion molecules in cells: adopting a migratory and invasive behavior [135]. Numerous DUBs are involved in cancer cell invasiveness through the regulation of different EMT transcription factors (Figure 4). 

SNAIL is a key regulator of EMT and plays an important role in tumor progression and metastasis. A group of DUBs, including OTUB1, DUB3, and USP3, are reported to stabilize Snail through preventing its ubiquitination and proteasomal degradation. OTUB1 promotes metastasis of esophageal squamous cell carcinoma through the stabilization of Snail [92]. DUB3 is found to be overexpressed in breast cancer, and depletion of DUB3 leads to Snail1 destabilization, which suppresses EMT, tumor invasiveness, and metastasis [90]. In addition, DUB3 also interacts with SLUG and TWIST and prevents their degradation, thereby promoting migration, invasion, and cancer stem cell-like properties in breast cancer cells [91]. Moreover, USP3 is significantly upregulated in glioblastomas and gastric cancer (GC). Clinicopathological data demonstrate that USP3 correlates with a shorter overall and relapse-free survival in glioblastomas [136]. It has also been reported that USP3 interacts with and stabilizes SUZ12 via deubiquitination. Expression of SUZ12 is negatively correlated with E-cadherin, which promotes migration and EMT in GC cells [95]. SMAD4 has been found to regulate EMT. USP17 is upregulated in osteosarcoma tissues and stabilizes SMAD4 through its DUB activity, leading to enhanced osteosarcoma cell invasion [94]. 

In addition to EMT mediators, DUBs target other molecules involved in cancer invasiveness. High expression of 14-3-3γ is found in various cancers, such as breast cancer and NSCLC [137,138]. Overexpression of 14-3-3γ promotes cell migration and invasion and correlates with the invasiveness of cancer cells. USP37 regulates the stability of 14-3-3γ through its DUB activity [97]. Another DUB, 26S proteasome non-ATPase regulatory subunit 14 (PSMD14), is a posttranslational regulator of growth factor receptor bound protein 2 (GRB2). PSMD14 is significantly upregulated in HCC tissues, and it inhibits the degradation of GRB2 via deubiquitination. Overexpression of PSMD14 correlates with vascular invasion, tumor recurrence, and poor tumor-free and overall survival in patients with HCC [93]. The small GTPase Ras-related protein RAB7 is an early-induced melanoma driver and endocytosis protein that favors tumor invasion [139]. It is suggested to play roles in modulating endosomal maturation and autophagosome resolution in various cell types [140,141]. It was recently shown to be regulated by USP32 [96].

## 8. DUBs as Therapeutic Targets for Cancer Treatment

As mentioned above, DUBs have been shown to deubiquitinate many targets involved in different characteristics of cancer (Table 2), suggesting that DUBs may be potential therapeutic targets in cancer treatment. Indeed, many studies have been conducted to examine the potential of DUBs in cancer therapeutics. As DUBs are part of the proteasome system, proteasome inhibitors target them, which has shown promising successes for cancer treatment. Several examples are given below. Bortezomib, the first proteasome inhibitor, has entered clinical practice to treat relapsed multiple myeloma and showed outstanding antimyeloma activity [142,143]. In addition, combination of bortezomib and epirubicin significantly increases the sensitivity of colorectal carcinoma cells to apoptosis [144]. Due to the resistance to bortezomib, next-generation proteasome inhibitors carfilzomib and ixazomib have been approved. Carfilzomib irreversibly binds to the β-5 subunit of the proteasome [145]. A preclinical study has demonstrated that carfilzomib increased efficacy against bortezomib-resistant multiple myeloma [146]. In the Phase 2 and Phase 3 clinical trials, single-agent carfilzomib provided durable anticancer activity in patients with relapsed and/or refractory multiple myeloma [147]. Ixazomib, the first oral proteasome inhibitor to enter the clinic, is now commonly used for multiple myeloma treatment. It is an efficacious and long-term therapy for patients with advanced stage multiple myeloma [148]. In a double-blind Phase 3 trial, the use of ixazomib significantly improved progression-free survival in patients with relapsed and/or refractory multiple myeloma [149].

In addition to proteasome inhibitors, numerous DUB therapeutic targets have been developed. One excellent and classical example is USP7. Activating p53 by inhibiting MDM2 is a major direction of cancer treatment [150,151]. Nutlin-3 from Roche and RITA (2,5-bis(5-hydroxymethyl-2-thienyl)furan (NSC652287)) from the National Cancer Institute have been developed for interfering with the MDM2/p53 interaction to induce p53 and therefore cell death in human tumor cells [152,153,154]. They represent an important class of small molecules that has significant antitumor effects without obvious toxicity in mice [153,155], which further suggests that promoting MDM2 degradation will provide a therapeutic benefit when treating p53-related cancers. Additionally, USP7 silencing promotes the degradation of MDM2 and thus abrogates p53 degradation. Targeting DUBs might provide a new direction for cancer treatment, as it has the advantage of a simpler mechanism than targeting UBQ ligases or the 26S proteasome [150,156]. A small molecule lead-like inhibitor of USP7, HBX41108, which stabilizes and activates p53, was identified using high-throughput screening [156]. This inhibitor symbolizes a milestone in DUB drug development and sheds light on new potential cancer therapies using DUB inhibitors. 

In addition, many cancer studies have focused on the apoptotic role of DUBs and exploited this role for chemotherapy. A drug screening study demonstrated that the small molecule DUB inhibitor b-AP15 inhibits two DUBs, USP14 and UCHL5. Treatment with b-AP15 results in apoptosis of human Waldenström macroglobulinemia (WM) cell lines and primary WM tumor cells [157]. In another chemotherapeutic study, pharmacological targeting of USP14 with the FDA-approved small-molecule inhibitor VLX1570 decreased viability in endometrial cancer cells through cell cycle arrest and caspase 3-mediated apoptosis [158]. The oncogenic transcription factor pre-B cell leukemia homeobox-1 (PBX1) promotes advanced PCa cell proliferation. USP9X interacts with and stabilizes the PBX1 protein by attenuating its Lys48-linked polyubiquitination. The USP9X inhibitor WP1130 markedly induces PBX1 degradation and promotes PCa cell apoptosis [159]. The selected DUB inhibitors that target on cancer cells are summarized in Table 3. To conclude, DUBs play multiple roles in cellular functions. The aberrant expression and regulation of these enzymes have been shown to contribute to promote tumorigenesis, making them promising therapeutic targets for cancer therapy.

## Figures and Tables

**Table 1:** The sub-cellular localizations of DUBs in mammalian cells.

Organelle	DUBs
Nucleolus	USP36, USP39
Nucleus	BAP1, MYSM1, USP1, USP11, USP22, USP26, USP28, USP29, USP3, USP42, USP44, USP49, USP51, USP7, USPL1, ZUP1
Golgi	USP32, USP33
Endoplasmic reticulum	ATXN3, USP13, USP19, USP33, YOD1
Microtubules	CYLD, USP21
Centriole	USP21, USP33, USP9X
Early endosome and multivesicular body	AMSH, AMSH-LP, USP2a, USP8
Lipid droplet	USP35
Peroxisome and mitochondrion	USP30
Cajal body	USPL1
Stress granule	USP10, USP13, USP5
Plasma membrane	JOSD1, USP6
Cytoplasm	A20, CYLD, PSMD14, UCHL5, USP14

**Table 2:** Functional roles of DUBs in cancer properties.

Functions	DUBs	Targets	References
Cell cycle control	BAP1	KLF5	[22]
	DUB3	cyclin A	[23]
	OTUD6B-2	cyclin D1 and c-Myc	[24]
	OTUD7B	APC/C, GβL, HIF2α and E2F1	[25,26,27,28]
	USP10	SKP2, Bcr-Abl	[29]
	USP14	AR	[30]
	USP17	p21, ELK-1, Su(var)3-9, Enhancer-of-zeste, and Trithorax domain-containing protein 8	[31,32,33]
	USP21	FOXM1	[34]
	USP3	KLF5	[35]
	USP7	PHF8	[36]
Cell proliferation	OTUB1	p53	[37]
	OTUD1	p53, SMAD7	[38,39]
	USP10	p53	[40]
	USP14	AR	[41]
	USP15	MDM2, TGF-β receptor	[42,43]
	USP2	MDM2	[44]
	USP28	p53, p21, and p16INK4a	[45,46]
	USP29	p53	[47]
	USP4	β-catenin, p53 and NF-κB	[48,49,50]
	USP42	P53	[45]
	USP49	FKBP51	[51]
	USP5	P53	[52]
	USP6NL	β-catenin	[53]
	USP7	MDM2	[54,55,56,57,58]
	USP9X	β-catenin, p53	[59,60]
Cell apoptosis	ATXN3	p53	[61]
	JOSD1	MCL1	[62]
	USP5	p53, MAF bZIP	[63,64]
DNA damage repair	BAP1	PR-DUB	[65]
	CYLD	p53	[66]
	OTUD5	SPT16	[67]
	OTUD7A	Rap80/BRCA1-A complex	[68]
	OTUD7B	Rap80/BRCA1-A complex	[68]
	UBP12	PCNA	[69]
	UBP2	PCNA	[69]
	UCHL5	NFRKB	[70]
	USP1	PCNA	[71,72,73,74]
	USP11	BRCA2	[75]
	UBP15	PCNA	[69]
	USP3	γH2AX and H2A	[76]
	USP48	BRCA1	[77]
	USP7	PHF8, pBmi1, Bmi1, RNF168, and BRCA1	[36,78]
	USP9X	claspin	[79]
Tumor suppression	CYLD	tumor necrosis factor receptor-associated factor 2, IKKγ	[80,81,82]
	USP11	PML	[83]
	USP13	PTEN	[84]
	USP46	PHLPP	[85]
Oncogene	BAP1	ASXL1	[86]
	USP22	c-Myc	[87]
	USP28	MYC	[88]
	USP9X	FBW7	[89]
Metastasis	DUB3	Snail, Slug and Twist	[90,91]
	OTUB1	Snail	[92]
	PSMD14	GRB2	[93]
	USP17	SMAD4	[94]
	USP3	SUZ12	[95]
	USP32	RAB7	[96]
	USP37	14-3-3γ	[97]

**Table 3:** Summary of known DUB inhibitors that are targeted in cancer cells.

DUBs	DUBs Inhibitors	Therapeutic Targets	Functional Effects	References
USP8	9-Ethyloxyimino-9H-indeno[1,2-b]pyrazine-2,3-dicarbonitrile	Non-small cell lung cancer	Downregulation of receptor tyrosine kinases including EGFR, ERBB2, ERBB3, and MET	[160]
UCHL1	LDN-57444	Lung cancer cell line	Inhibit proliferation	[161]
UCHL1, UCHL3	TCID	Multiple myeloma	Induce apoptosis	[162]
USP1	Pimozide	Leukemic cell lines	Promoted the degradation of ID1	[163]
USP1-UAF1	ML323	Non-small cell lung cancer and osteosarcoma cells	Induced DNA damage	[164]
USP1-UAF1	Pimozide and GW7647	Non–small cell lung cancer	Inhibit cell proliferation	[165]
USP2	ML346	Colorectal cancer nad mantle cell lymphoma	Accelerate cyclin D1 degradation, cell cycle arrest	[166]
USP2a/USP2b/USP5/USP8	AM146, RA-9 and RA-14	Breast, ovarian and cervical cancer cell lines	Downregulation cell-cycle promoter, and upregulation of tumor suppressor	[167]
USP5/IsoT, USP4	Vialinin A	Basophilic leukemia cells	Inhibit the release of TNFα	[168]
USP7	HBX 41,108	Colorectal carcinoma	Induced p53-dependent apoptosis	[156]
USP7/USP47	P5091 and Compound 1	Multiple myeloma	Induce apoptosis, inhibit tumor growth	[169,170]
USP9X/USP5/USP24	WP1130	Mantle cell lymphoma	Downregulation of antiapoptotic and upregulation of proapoptotic proteins, such as MCL-1 and p53	[171,172]
USP14/ UCHL5	AC17	Human lung cancer cells	Inhibit NFκB pathway and reactive p53	[173]
USP14/UCHL5	b-AP15 (WO2013058691)	Multiple myeloma/ colorectal carcinoma	Downregulation of CDC25C, CDC2, and cyclin B1/ overexpression of the anti-apoptotic mediator Bcl-2 and anti-tumor activity	[162,174]
USP14/UCHL5	VLX1570	Colon carcinoma cell	Inhibit proteasome DUB activity	[175]

## Abbreviations

53BP1	p53-binding protein 1
Akt	protein kinase B
APC/C	anaphase-promoting complex/cyclosome
AR	androgen receptor
BAP1	BRCA1 associated protein 1
BRCA	breast-cancer susceptibility gene
BRCC3	BRCA1/BRCA2-containing complex 3
CDK	cyclin-dependent kinase
CRC	colorectal cancer
DDR	DNA damage response
DSB	double-strand break
DUB	Deubiquitinase
ELK-1	ETS like-1 protein
EMT	epithelial-mesenchymal transition
FBW7	F-box and WD repeat domain-containing 7
FKBP51	FK506-binding protein 51
GC	gastric cancer
GRB2	growth factor receptor bound protein 2
HCC	hepatocellular carcinoma
JOSD1	Josephin domain containing 1
KLF5	Krüppel-like factor 5
MDM2	mouse double minute 2
mTORC1	mammalian target of rapamycin complex 1
NK-κB	nuclear factor kappa-light-chain-enhancer of activated B cells
NSCLC	non-small cell lung cancer
OTUB1	otubain 1
OTU	otubain protease
PBX1	pre-B cell leukemia homeobox-1
PCa	prostate cancer
PCNA	proliferating-cell nuclear antigen
PHF8	PHD finger protein 8
PHLPP	PH domain leucine-rich-repeats protein phosphatase
PML	promyelocytic leukemia
PSMD14	26S proteasome non-ATPase regulatory subunit 14
PTEN	phosphatase and tensin homolog deleted on chromosome 10
RNF	ring finger proteins
SKP2	S-phase kinase associated protein 2.
TGF-β	transforming growth factor beta
TRAIL	tumor necrosis factor alpha apoptosis-inducing ligand
UBQ	ubiquitin
UBR5	ubiquitin protein ligase E3 component N-recognin 5
UCH	ubiquitin C-terminal hydrolases
UCHL	ubiquitin C-terminal hydrolases like
USP	ubiquitin-specific protease
WM	Waldenström macroglobulinemia

## References

[1] Nijman S.M.B., Luna-Vargas M.P.A., Velds A., Brummelkamp T.R., Dirac A.M.G., Sixma T.K., Bernards R. (2005). A genomic and functional inventory of deubiquitinating enzymes. Cell.

[2] Abdul Rehman S.A., Kristariyanto Y.A., Choi S.Y., Nkosi P.J., Weidlich S., Labib K., Hofmann K., Kulathu Y. (2016). Mindy-1 is a member of an evolutionarily conserved and structurally distinct new family of deubiquitinating enzymes. Mol. Cell.

[3] Liang J., Saad Y., Lei T., Wang J., Qi D., Yang Q., Kolattukudy P.E., Fu M. (2010). Mcp-induced protein 1 deubiquitinates traf proteins and negatively regulates jnk and nf-kappab signaling. J. Exp. Med..

[4] Hermanns T., Pichlo C., Woiwode I., Klopffleisch K., Witting K.F., Ovaa H., Baumann U., Hofmann K. (2018). A family of unconventional deubiquitinases with modular chain specificity determinants. Nat. Commun..

[5] Clague M.J., Barsukov I., Coulson J.M., Liu H., Rigden D.J., Urbe S. (2013). Deubiquitylases from genes to organism. Physiol. Rev..

[6] Sowa M.E., Bennett E.J., Gygi S.P., Harper J.W. (2009). Defining the human deubiquitinating enzyme interaction landscape. Cell.

[7] Komander D., Clague M.J., Urbe S. (2009). Breaking the chains: Structure and function of the deubiquitinases. Nat. Rev. Mol. Cell Biol..

[8] Urbe S., Liu H., Hayes S.D., Heride C., Rigden D.J., Clague M.J. (2012). Systematic survey of deubiquitinase localization identifies usp21 as a regulator of centrosome- and microtubule-associated functions. Mol. Biol Cell.

[9] Hershko A., Ciechanover A. (1998). The ubiquitin system. Annu. Rev. Biochem..

[10] Pickart C.M. (2001). Ubiquitin enters the new millennium. Mol. Cell.

[11] Ventii K.H., Wilkinson K.D. (2008). Protein partners of deubiquitinating enzymes. Biochem. J..

[12] Mennerich D., Kubaichuk K., Kietzmann T. (2019). Dubs, hypoxia, and cancer. Trends Cancer.

[13] He M., Zhou Z., Wu G., Chen Q., Wan Y. (2017). Emerging role of dubs in tumor metastasis and apoptosis: Therapeutic implication. Pharmacol. Ther..

[14] Hussain S., Zhang Y., Galardy P.J. (2009). Dubs and cancer: The role of deubiquitinating enzymes as oncogenes, non-oncogenes and tumor suppressors. Cell Cycle.

[15] Bednash J.S., Mallampalli R.K. (2018). Targeting deubiquitinases in cancer. Methods Mol. Biol..

[16] Fraile J.M., Quesada V., Rodriguez D., Freije J.M., Lopez-Otin C. (2012). Deubiquitinases in cancer: New functions and therapeutic options. Oncogene.

[17] Cheng J., Guo J., North B.J., Wang B., Cui C.P., Li H., Tao K., Zhang L., Wei W. (2019). Functional analysis of deubiquitylating enzymes in tumorigenesis and development. Biochim. Et Biophys. Acta. Rev. Cancer.

[18] Clague M.J., Urbe S., Komander D. (2019). Breaking the chains: Deubiquitylating enzyme specificity begets function. Nat. Rev. Mol. Cell Biol..

[19] Venuto S., Merla G. (2019). E3 ubiquitin ligase trim proteins, cell cycle and mitosis. Cells.

[20] Vodermaier H.C. (2004). Apc/c and scf: Controlling each other and the cell cycle. Curr. Biol..

[21] Sivakumar S., Gorbsky G.J. (2015). Spatiotemporal regulation of the anaphase-promoting complex in mitosis. Nat. Rev. Mol. Cell Biol..

[22] Qin J., Zhou Z., Chen W., Wang C., Zhang H., Ge G., Shao M., You D., Fan Z., Xia H. (2015). Bap1 promotes breast cancer cell proliferation and metastasis by deubiquitinating klf5. Nat. Commun..

[23] Hu B., Deng T., Ma H., Liu Y., Feng P., Wei D., Ling N., Li L., Qiu S., Zhang L. (2019). Deubiquitinase dub3 regulates cell cycle progression via stabilizing cyclin a for proliferation of non-small cell lung cancer cells. Cells.

[24] Sobol A., Askonas C., Alani S., Weber M.J., Ananthanarayanan V., Osipo C., Bocchetta M. (2017). Deubiquitinase otud6b isoforms are important regulators of growth and proliferation. Mol. Cancer Res..

[25] Bonacci T., Suzuki A., Grant G.D., Stanley N., Cook J.G., Brown N.G., Emanuele M.J. (2018). Cezanne/otud7b is a cell cycle-regulated deubiquitinase that antagonizes the degradation of apc/c substrates. EMBO J..

[26] Bonacci T., Emanuele M.J. (2019). Impressionist portraits of mitotic exit: Apc/c, k11-linked ubiquitin chains and cezanne. Cell Cycle.

[27] Moniz S., Bandarra D., Biddlestone J., Campbell K.J., Komander D., Bremm A., Rocha S. (2015). Cezanne regulates e2f1-dependent hif2alpha expression. J. Cell Sci..

[28] Wang B., Jie Z., Joo D., Ordureau A., Liu P., Gan W., Guo J., Zhang J., North B.J., Dai X. (2017). Traf2 and otud7b govern a ubiquitin-dependent switch that regulates mtorc2 signalling. Nature.

[29] Liao Y., Liu N., Xia X., Guo Z., Li Y., Jiang L., Zhou R., Tang D., Huang H., Liu J. (2019). Usp10 modulates the skp2/bcr-abl axis via stabilizing skp2 in chronic myeloid leukemia. Cell Discov..

[30] Liao Y., Xia X., Liu N., Cai J., Guo Z., Li Y., Jiang L., Dou Q.P., Tang D., Huang H. (2018). Growth arrest and apoptosis induction in androgen receptor-positive human breast cancer cells by inhibition of usp14-mediated androgen receptor deubiquitination. Oncogene.

[31] McFarlane C., Kelvin A.A., de la Vega M., Govender U., Scott C.J., Burrows J.F., Johnston J.A. (2010). The deubiquitinating enzyme usp17 is highly expressed in tumor biopsies, is cell cycle regulated, and is required for g1-s progression. Cancer Res..

[32] Ducker C., Chow L.K.Y., Saxton J., Handwerger J., McGregor A., Strahl T., Layfield R., Shaw P.E. (2019). De-ubiquitination of elk-1 by usp17 potentiates mitogenic gene expression and cell proliferation. Nucleic Acids Res..

[33] Fukuura K., Inoue Y., Miyajima C., Watanabe S., Tokugawa M., Morishita D., Ohoka N., Komada M., Hayashi H. (2019). The ubiquitin-specific protease usp17 prevents cellular senescence by stabilizing the methyltransferase set8 and transcriptionally repressing p21. J. Biol. Chem..

[34] Arceci A., Bonacci T., Wang X., Stewart K., Damrauer J.S., Hoadley K.A., Emanuele M.J. (2019). Foxm1 deubiquitination by usp21 regulates cell cycle progression and paclitaxel sensitivity in basal-like breast cancer. Cell Rep..

[35] Wu Y., Qin J., Li F., Yang C., Li Z., Zhou Z., Zhang H., Li Y., Wang X., Liu R. (2019). Usp3 promotes breast cancer cell proliferation by deubiquitinating klf5. J. Biol. Chem..

[36] Wang Q., Ma S., Song N., Li X., Liu L., Yang S., Ding X., Shan L., Zhou X., Su D. (2016). Stabilization of histone demethylase phf8 by usp7 promotes breast carcinogenesis. J. Clin. Investig..

[37] Saldana M., VanderVorst K., Berg A.L., Lee H., Carraway K.L. (2019). Otubain 1: A non-canonical deubiquitinase with an emerging role in cancer. Endocr. Relat. Cancer.

[38] Piao S., Pei H.Z., Huang B., Baek S.H. (2017). Ovarian tumor domain-containing protein 1 deubiquitinates and stabilizes p53. Cell. Signal..

[39] Zhang Z., Fan Y., Xie F., Zhou H., Jin K., Shao L., Shi W., Fang P., Yang B., van Dam H. (2017). Breast cancer metastasis suppressor otud1 deubiquitinates smad7. Nat. Commun..

[40] Yuan J., Luo K., Zhang L., Cheville J.C., Lou Z. (2010). Usp10 regulates p53 localization and stability by deubiquitinating p53. Cell.

[41] Liao Y., Liu N., Hua X., Cai J., Xia X., Wang X., Huang H., Liu J. (2017). Proteasome-associated deubiquitinase ubiquitin-specific protease 14 regulates prostate cancer proliferation by deubiquitinating and stabilizing androgen receptor. Cell Death Dis..

[42] Zou Q., Jin J., Hu H., Li H.S., Romano S., Xiao Y., Nakaya M., Zhou X., Cheng X., Yang P. (2014). Usp15 stabilizes mdm2 to mediate cancer-cell survival and inhibit antitumor t cell responses. Nat. Immunol..

[43] Eichhorn P.J., Rodon L., Gonzalez-Junca A., Dirac A., Gili M., Martinez-Saez E., Aura C., Barba I., Peg V., Prat A. (2012). Usp15 stabilizes tgf-beta receptor i and promotes oncogenesis through the activation of tgf-beta signaling in glioblastoma. Nat. Med..

[44] Stevenson L.F., Sparks A., Allende-Vega N., Xirodimas D.P., Lane D.P., Saville M.K. (2007). The deubiquitinating enzyme usp2a regulates the p53 pathway by targeting mdm2. EMBO J..

[45] Hock A.K., Vigneron A.M., Carter S., Ludwig R.L., Vousden K.H. (2011). Regulation of p53 stability and function by the deubiquitinating enzyme usp42. EMBO J..

[46] Cuella-Martin R., Oliveira C., Lockstone H.E., Snellenberg S., Grolmusova N., Chapman J.R. (2016). 53bp1 integrates DNA repair and p53-dependent cell fate decisions via distinct mechanisms. Mol. Cell.

[47] Liu J., Chung H.J., Vogt M., Jin Y., Malide D., He L., Dundr M., Levens D. (2011). Jtv1 co-activates fbp to induce usp29 transcription and stabilize p53 in response to oxidative stress. EMBO J..

[48] Yun S.I., Kim H.H., Yoon J.H., Park W.S., Hahn M.J., Kim H.C., Chung C.H., Kim K.K. (2015). Ubiquitin specific protease 4 positively regulates the wnt/beta-catenin signaling in colorectal cancer. Mol. Oncol..

[49] Li Z., Hao Q., Luo J., Xiong J., Zhang S., Wang T., Bai L., Wang W., Chen M., Wang W. (2016). Usp4 inhibits p53 and nf-κb through deubiquitinating and stabilizing hdac2. Oncogene.

[50] Zhang X., Berger F.G., Yang J., Lu X. (2011). Usp4 inhibits p53 through deubiquitinating and stabilizing arf-bp1. EMBO J..

[51] Luo K., Li Y., Yin Y., Li L., Wu C., Chen Y., Nowsheen S., Hu Q., Zhang L., Lou Z. (2017). Usp49 negatively regulates tumorigenesis and chemoresistance through fkbp51-akt signaling. EMBO J..

[52] Potu H., Peterson L.F., Pal A., Verhaegen M., Cao J., Talpaz M., Donato N.J. (2014). Usp5 links suppression of p53 and fas levels in melanoma to the braf pathway. Oncotarget.

[53] Sun K., He S.B., Yao Y.Z., Qu J.G., Xie R., Ma Y.Q., Zong M.H., Chen J.X. (2019). Tre2 (usp6nl) promotes colorectal cancer cell proliferation via wnt/beta-catenin pathway. Cancer Cell Int..

[54] Cummins J.M., Vogelstein B. (2004). Hausp is required for p53 destabilization. Cell Cycle.

[55] Li M., Brooks C.L., Kon N., Gu W. (2004). A dynamic role of hausp in the p53-mdm2 pathway. Mol. Cell.

[56] Hu M., Gu L., Li M., Jeffrey P.D., Gu W., Shi Y. (2006). Structural basis of competitive recognition of p53 and mdm2 by hausp/usp7: Implications for the regulation of the p53-mdm2 pathway. PLoS Biol..

[57] Sheng Y., Saridakis V., Sarkari F., Duan S., Wu T., Arrowsmith C.H., Frappier L. (2006). Molecular recognition of p53 and mdm2 by usp7/hausp. Nat. Struct. Mol. Biol..

[58] Li M., Chen D., Shiloh A., Luo J., Nikolaev A.Y., Qin J., Gu W. (2002). Deubiquitination of p53 by hausp is an important pathway for p53 stabilization. Nature.

[59] Yang B., Zhang S., Wang Z., Yang C., Ouyang W., Zhou F., Zhou Y., Xie C. (2016). Deubiquitinase usp9x deubiquitinates beta-catenin and promotes high grade glioma cell growth. Oncotarget.

[60] Liu H., Chen W., Liang C., Chen B.W., Zhi X., Zhang S., Zheng X., Bai X., Liang T. (2015). Wp1130 increases doxorubicin sensitivity in hepatocellular carcinoma cells through usp9x-dependent p53 degradation. Cancer Lett..

[61] Liu H., Li X., Ning G., Zhu S., Ma X., Liu X., Liu C., Huang M., Schmitt I., Wullner U. (2016). The machado-joseph disease deubiquitinase ataxin-3 regulates the stability and apoptotic function of p53. PLoS Biol..

[62] Wu X., Luo Q., Zhao P., Chang W., Wang Y., Shu T., Ding F., Li B., Liu Z. (2020). Josd1 inhibits mitochondrial apoptotic signalling to drive acquired chemoresistance in gynaecological cancer by stabilizing mcl1. Cell Death Differ..

[63] Potu H., Kandarpa M., Peterson L.F., Donato N.J., Talpaz M. (2019). Tumor necrosis factor related apoptosis inducing ligand (trail) regulates deubiquitinase usp5 in tumor cells. Oncotarget.

[64] Wang S., Juan J., Zhang Z., Du Y., Xu Y., Tong J., Cao B., Moran M.F., Zeng Y., Mao X. (2017). Inhibition of the deubiquitinase usp5 leads to c-maf protein degradation and myeloma cell apoptosis. Cell Death Dis..

[65] Ismail I.H., Davidson R., Gagne J.P., Xu Z.Z., Poirier G.G., Hendzel M.J. (2014). Germline mutations in bap1 impair its function in DNA double-strand break repair. Cancer Res..

[66] Fernandez-Majada V., Welz P.S., Ermolaeva M.A., Schell M., Adam A., Dietlein F., Komander D., Buttner R., Thomas R.K., Schumacher B. (2016). The tumour suppressor cyld regulates the p53 DNA damage response. Nat. Commun..

[67] de Vivo A., Sanchez A., Yegres J., Kim J., Emly S., Kee Y. (2019). The otud5-ubr5 complex regulates fact-mediated transcription at damaged chromatin. Nucleic Acids Res..

[68] Wu X., Liu S., Sagum C., Chen J., Singh R., Chaturvedi A., Horton J.R., Kashyap T.R., Fushman D., Cheng X. (2019). Crosstalk between lys63- and lys11-polyubiquitin signaling at DNA damage sites is driven by cezanne. Genes Dev..

[69] Alvarez V., Vinas L., Gallego-Sanchez A., Andres S., Sacristan M.P., Bueno A. (2016). Orderly progression through s-phase requires dynamic ubiquitylation and deubiquitylation of pcna. Sci. Rep..

[70] Nishi R., Wijnhoven P., le Sage C., Tjeertes J., Galanty Y., Forment J.V., Clague M.J., Urbe S., Jackson S.P. (2014). Systematic characterization of deubiquitylating enzymes for roles in maintaining genome integrity. Nat. Cell Biol.

[71] Whitehurst C.B., Vaziri C., Shackelford J., Pagano J.S. (2012). Epstein-barr virus bplf1 deubiquitinates pcna and attenuates polymerase eta recruitment to DNA damage sites. J. Virol..

[72] Castella M., Jacquemont C., Thompson E.L., Yeo J.E., Cheung R.S., Huang J.W., Sobeck A., Hendrickson E.A., Taniguchi T. (2015). Fanci regulates recruitment of the fa core complex at sites of DNA damage independently of fancd2. PLoS Genet..

[73] Nijman S.M., Huang T.T., Dirac A.M., Brummelkamp T.R., Kerkhoven R.M., D’Andrea A.D., Bernards R. (2005). The deubiquitinating enzyme usp1 regulates the fanconi anemia pathway. Mol. Cell.

[74] Olazabal-Herrero A., Garcia-Santisteban I., Rodriguez J.A. (2015). Structure-function analysis of usp1: Insights into the role of ser313 phosphorylation site and the effect of cancer-associated mutations on autocleavage. Mol. Cancer.

[75] Orthwein A., Noordermeer S.M., Wilson M.D., Landry S., Enchev R.I., Sherker A., Munro M., Pinder J., Salsman J., Dellaire G. (2015). A mechanism for the suppression of homologous recombination in g1 cells. Nature.

[76] Sharma N., Zhu Q., Wani G., He J., Wang Q.E., Wani A.A. (2014). Usp3 counteracts rnf168 via deubiquitinating h2a and gammah2ax at lysine 13 and 15. Cell Cycle.

[77] Uckelmann M., Densham R.M., Baas R., Winterwerp H.H.K., Fish A., Sixma T.K., Morris J.R. (2018). Usp48 restrains resection by site-specific cleavage of the brca1 ubiquitin mark from h2a. Nat. Commun..

[78] Zhu Q., Sharma N., He J., Wani G., Wani A.A. (2015). Usp7 deubiquitinase promotes ubiquitin-dependent DNA damage signaling by stabilizing rnf168. Cell Cycle.

[79] McGarry E., Gaboriau D., Rainey M.D., Restuccia U., Bachi A., Santocanale C. (2016). The deubiquitinase usp9x maintains DNA replication fork stability and DNA damage checkpoint responses by regulating claspin during s-phase. Cancer Res..

[80] Kovalenko A., Chable-Bessia C., Cantarella G., Israel A., Wallach D., Courtois G. (2003). The tumour suppressor cyld negatively regulates nf-kappa b signalling by deubiquitination. Nature.

[81] Trompouki E., Hatzivassiliou E., Tsichritzis T., Farmer H., Ashworth A., Mosialos G. (2003). Cyld is a deubiquitinating enzyme that negatively regulates nf-kappa b activation by tnfr family members. Nature.

[82] Brummelkamp T.R., Nijman S.M., Dirac A.M., Bernards R. (2003). Loss of the cylindromatosis tumour suppressor inhibits apoptosis by activating nf-kappab. Nature.

[83] Wu H.C., Lin Y.C., Liu C.H., Chung H.C., Wang Y.T., Lin Y.W., Ma H.I., Tu P.H., Lawler S.E., Chen R.H. (2014). Usp11 regulates pml stability to control notch-induced malignancy in brain tumours. Nat. Commun..

[84] Qu Z., Zhang R., Su M., Liu W. (2019). Usp13 serves as a tumor suppressor via the pten/akt pathway in oral squamous cell carcinoma. Cancer Manag. Res..

[85] Li X., Stevens P.D., Yang H., Gulhati P., Wang W., Evers B.M., Gao T. (2013). The deubiquitination enzyme usp46 functions as a tumor suppressor by controlling phlpp-dependent attenuation of akt signaling in colon cancer. Oncogene.

[86] Bott M., Brevet M., Taylor B.S., Shimizu S., Ito T., Wang L., Creaney J., Lake R.A., Zakowski M.F., Reva B. (2011). The nuclear deubiquitinase bap1 is commonly inactivated by somatic mutations and 3p21.1 losses in malignant pleural mesothelioma. Nat. Genet..

[87] Kim D., Hong A., Park H.I., Shin W.H., Yoo L., Jeon S.J., Chung K.C. (2017). Deubiquitinating enzyme usp22 positively regulates c-myc stability and tumorigenic activity in mammalian and breast cancer cells. J. Cell Physiol..

[88] Diefenbacher M.E., Popov N., Blake S.M., Schulein-Volk C., Nye E., Spencer-Dene B., Jaenicke L.A., Eilers M., Behrens A. (2014). The deubiquitinase usp28 controls intestinal homeostasis and promotes colorectal cancer. J. Clin. Inv..

[89] Khan O.M., Carvalho J., Spencer-Dene B., Mitter R., Frith D., Snijders A.P., Wood S.A., Behrens A. (2018). The deubiquitinase usp9x regulates fbw7 stability and suppresses colorectal cancer. J. Clin. Inv..

[90] Wu Y., Wang Y., Lin Y., Liu Y., Wang Y., Jia J., Singh P., Chi Y.I., Wang C., Dong C. (2017). Dub3 inhibition suppresses breast cancer invasion and metastasis by promoting snail1 degradation. Nat. Commun..

[91] Lin Y., Wang Y., Shi Q., Yu Q., Liu C., Feng J., Deng J., Evers B.M., Zhou B.P., Wu Y. (2017). Stabilization of the transcription factors slug and twist by the deubiquitinase dub3 is a key requirement for tumor metastasis. Oncotarget.

[92] Zhou H., Liu Y., Zhu R., Ding F., Cao X., Lin D., Liu Z. (2018). Otub1 promotes esophageal squamous cell carcinoma metastasis through modulating snail stability. Oncogene.

[93] Lv J., Zhang S., Wu H., Lu J., Lu Y., Wang F., Zhao W., Zhan P., Lu J., Fang Q. (2020). Deubiquitinase psmd14 enhances hepatocellular carcinoma growth and metastasis by stabilizing grb2. Cancer lett..

[94] Song C., Liu W., Li J. (2017). Usp17 is upregulated in osteosarcoma and promotes cell proliferation, metastasis, and epithelial-mesenchymal transition through stabilizing smad4. Tumour Biol..

[95] Wu X., Liu M., Zhu H., Wang J., Dai W., Li J., Zhu D., Tang W., Xiao Y., Lin J. (2019). Ubiquitin-specific protease 3 promotes cell migration and invasion by interacting with and deubiquitinating suz12 in gastric cancer. J. Exp. Clin. Cancer Res..

[96] Sapmaz A., Berlin I., Bos E., Wijdeven R.H., Janssen H., Konietzny R., Akkermans J.J., Erson-Bensan A.E., Koning R.I., Kessler B.M. (2019). Usp32 regulates late endosomal transport and recycling through deubiquitylation of rab7. Nat. Commun..

[97] Kim J.O., Kim S.R., Lim K.H., Kim J.H., Ajjappala B., Lee H.J., Choi J.I., Baek K.H. (2015). Deubiquitinating enzyme usp37 regulating oncogenic function of 14-3-3gamma. Oncotarget.

[98] Yuan J., Luo K., Deng M., Li Y., Yin P., Gao B., Fang Y., Wu P., Liu T., Lou Z. (2014). Herc2-usp20 axis regulates DNA damage checkpoint through claspin. Nucleic Acids Res..

[99] Jiang Y.Z., Ma D., Suo C., Shi J., Xue M., Hu X., Xiao Y., Yu K.D., Liu Y.R., Yu Y. (2019). Genomic and transcriptomic landscape of triple-negative breast cancers: Subtypes and treatment strategies. Cancer Cell.

[100] Chiu H.W., Lin H.Y., Tseng I.J., Lin Y.F. (2018). Otud7b upregulation predicts a poor response to paclitaxel in patients with triple-negative breast cancer. Oncotarget.

[101] Lin D.D., Shen Y., Qiao S., Liu W.W., Zheng L., Wang Y.N., Cui N., Wang Y.F., Zhao S., Shi J.H. (2019). Upregulation of otud7b (cezanne) promotes tumor progression via akt/vegf pathway in lung squamous carcinoma and adenocarcinoma. Front. Oncol..

[102] Anastas J.N., Zee B.M., Kalin J.H., Kim M., Guo R., Alexandrescu S., Blanco M.A., Giera S., Gillespie S.M., Das J. (2019). Re-programing chromatin with a bifunctional lsd1/hdac inhibitor induces therapeutic differentiation in dipg. Cancer Cell.

[103] Zhan T., Rindtorff N., Boutros M. (2017). Wnt signaling in cancer. Oncogene.

[104] Ben-Porath I., Thomson M.W., Carey V.J., Ge R., Bell G.W., Regev A., Weinberg R.A. (2008). An embryonic stem cell-like gene expression signature in poorly differentiated aggressive human tumors. Nat. Genet..

[105] Reya T., Morrison S.J., Clarke M.F., Weissman I.L. (2001). Stem cells, cancer, and cancer stem cells. Nature.

[106] Nguyen H.H., Kim T., Nguyen T., Hahn M.J., Yun S.I., Kim K.K. (2019). A selective inhibitor of ubiquitin-specific protease 4 suppresses colorectal cancer progression by regulating beta-catenin signaling. Cell. Physiol. Biochem.: Int. J. Exp. Cell. Physiol. Biochem. Pharmacol..

[107] Brooks C.L., Gu W. (2011). P53 regulation by ubiquitin. FEBS Lett..

[108] Brooks C.L., Li M., Hu M., Shi Y., Gu W. (2007). The p53--mdm2--hausp complex is involved in p53 stabilization by hausp. Oncogene.

[109] Li F., Han H., Sun Q., Liu K., Lin N., Xu C., Zhao Z., Zhao W. (2019). Usp28 regulates deubiquitination of histone h2a and cell proliferation. Exp. Cell Res..

[110] Niendorf S., Oksche A., Kisser A., Lohler J., Prinz M., Schorle H., Feller S., Lewitzky M., Horak I., Knobeloch K.P. (2007). Essential role of ubiquitin-specific protease 8 for receptor tyrosine kinase stability and endocytic trafficking in vivo. Mol. Cell Biol..

[111] Hanahan D., Weinberg R.A. (2000). The hallmarks of cancer. Cell.

[112] Fernald K., Kurokawa M. (2013). Evading apoptosis in cancer. Trends Cell Biol..

[113] Burrows J.F., McGrattan M.J., Rascle A., Humbert M., Baek K.H., Johnston J.A. (2004). Dub-3, a cytokine-inducible deubiquitinating enzyme that blocks proliferation. J. Biol. Chem..

[114] Xu M., Takanashi M., Oikawa K., Tanaka M., Nishi H., Isaka K., Kudo M., Kuroda M. (2009). Usp15 plays an essential role for caspase-3 activation during paclitaxel-induced apoptosis. Biochem Biophys Res. Commun.

[115] Jin Z., Li Y., Pitti R., Lawrence D., Pham V.C., Lill J.R., Ashkenazi A. (2009). Cullin3-based polyubiquitination and p62-dependent aggregation of caspase-8 mediate extrinsic apoptosis signaling. Cell.

[116] Crook N.E., Clem R.J., Miller L.K. (1993). An apoptosis-inhibiting baculovirus gene with a zinc finger-like motif. J. Virol..

[117] Mei Y., Hahn A.A., Hu S., Yang X. (2011). The usp19 deubiquitinase regulates the stability of c-iap1 and c-iap2. J. Biol. Chem..

[118] Goncharov T., Niessen K., de Almagro M.C., Izrael-Tomasevic A., Fedorova A.V., Varfolomeev E., Arnott D., Deshayes K., Kirkpatrick D.S., Vucic D. (2013). Otub1 modulates c-iap1 stability to regulate signalling pathways. EMBO J..

[119] Engel K., Rudelius M., Slawska J., Jacobs L., Ahangarian Abhari B., Altmann B., Kurutz J., Rathakrishnan A., Fernandez-Saiz V., Brunner A. (2016). Usp9x stabilizes xiap to regulate mitotic cell death and chemoresistance in aggressive b-cell lymphoma. EMBO Mol. Med..

[120] Weber A., Heinlein M., Dengjel J., Alber C., Singh P.K., Hacker G. (2016). The deubiquitinase usp27x stabilizes the bh3-only protein bim and enhances apoptosis. EMBO Rep..

[121] Ghosal G., Chen J. (2013). DNA damage tolerance: A double-edged sword guarding the genome. Transl. Cancer Res..

[122] Hoeijmakers J.H. (2001). Genome maintenance mechanisms for preventing cancer. Nature.

[123] Le J., Perez E., Nemzow L., Gong F. (2019). Role of deubiquitinases in DNA damage response. DNA Repair.

[124] Jackson S.P., Bartek J. (2009). The DNA-damage response in human biology and disease. Nature.

[125] Shanmugam I., Abbas M., Ayoub F., Mirabal S., Bsaili M., Caulder E.K., Weinstock D.M., Tomkinson A.E., Hromas R., Shaheen M. (2014). Ubiquitin-specific peptidase 20 regulates rad17 stability, checkpoint kinase 1 phosphorylation and DNA repair by homologous recombination. J. Biol. Chem..

[126] Yoshida K., Miki Y. (2004). Role of brca1 and brca2 as regulators of DNA repair, transcription, and cell cycle in response to DNA damage. Cancer Sci..

[127] Meyer T., Jahn N., Lindner S., Rohner L., Dolnik A., Weber D., Scheffold A., Kopff S., Paschka P., Gaidzik V.I. (2020). Functional characterization of brcc3 mutations in acute myeloid leukemia with t(8;21)(q22;q22.1). Leukemia.

[128] Lancini C., van den Berk P.C., Vissers J.H., Gargiulo G., Song J.Y., Hulsman D., Serresi M., Tanger E., Blom M., Vens C. (2014). Tight regulation of ubiquitin-mediated DNA damage response by usp3 preserves the functional integrity of hematopoietic stem cells. J. Exp. Med..

[129] Bignell G.R., Warren W., Seal S., Takahashi M., Rapley E., Barfoot R., Green H., Brown C., Biggs P.J., Lakhani S.R. (2000). Identification of the familial cylindromatosis tumour-suppressor gene. Nat. Genet..

[130] Strobel P., Zettl A., Ren Z., Starostik P., Riedmiller H., Storkel S., Muller-Hermelink H.K., Marx A. (2002). Spiradenocylindroma of the kidney: Clinical and genetic findings suggesting a role of somatic mutation of the cyld1 gene in the oncogenesis of an unusual renal neoplasm. Am. J. Surg. Pathol..

[131] Hirai Y., Kawamata Y., Takeshima N., Furuta R., Kitagawa T., Kawaguchi T., Hasumi K., Sugai S., Noda T. (2004). Conventional and array-based comparative genomic hybridization analyses of novel cell lines harboring hpv18 from glassy cell carcinoma of the uterine cervix. Int. J. Oncol..

[132] Reiley W., Zhang M.Y., Sun S.C. (2004). Negative regulation of jnk signaling by the tumor suppressor cyld. J. Biol. Chem..

[133] Ikeda F., Dikic I. (2006). Cyld in ubiquitin signaling and tumor pathogenesis. Cell.

[134] Wang Z., Inuzuka H., Fukushima H., Wan L., Gao D., Shaik S., Sarkar F.H., Wei W. (2011). Emerging roles of the fbw7 tumour suppressor in stem cell differentiation. EMBO Rep..

[135] Nieto M.A., Huang R.Y., Jackson R.A., Thiery J.P. (2016). Emt: 2016. Cell.

[136] Fan L., Chen Z., Wu X., Cai X., Feng S., Lu J., Wang H., Liu N. (2019). Ubiquitin-specific protease 3 promotes glioblastoma cell invasion and epithelial-mesenchymal transition via stabilizing snail. Mol. Cancer Res. MCR.

[137] Hiraoka E., Mimae T., Ito M., Kadoya T., Miyata Y., Ito A., Okada M. (2019). Correction to: Breast cancer cell motility is promoted by 14-3-3gamma. Breast Cancer.

[138] Raungrut P., Wongkotsila A., Champoochana N., Lirdprapamongkol K., Svasti J., Thongsuksai P. (2018). Knockdown of 14-3-3gamma suppresses epithelial-mesenchymal transition and reduces metastatic potential of human non-small cell lung cancer cells. Anticancer Res..

[139] Alonso-Curbelo D., Riveiro-Falkenbach E., Perez-Guijarro E., Cifdaloz M., Karras P., Osterloh L., Megias D., Canon E., Calvo T.G., Olmeda D. (2014). Rab7 controls melanoma progression by exploiting a lineage-specific wiring of the endolysosomal pathway. Cancer Cell.

[140] Wang T., Ming Z., Xiaochun W., Hong W. (2011). Rab7: Role of its protein interaction cascades in endo-lysosomal traffic. Cell. Sign..

[141] Zhang M., Chen L., Wang S., Wang T. (2009). Rab7: Roles in membrane trafficking and disease. Biosci. Rep..

[142] Richardson P.G., Barlogie B., Berenson J., Singhal S., Jagannath S., Irwin D., Rajkumar S.V., Srkalovic G., Alsina M., Alexanian R. (2003). A phase 2 study of bortezomib in relapsed, refractory myeloma. N. Engl. J. Med..

[143] Richardson P.G., Sonneveld P., Schuster M.W., Irwin D., Stadtmauer E.A., Facon T., Harousseau J.L., Ben-Yehuda D., Lonial S., Goldschmidt H. (2005). Bortezomib or high-dose dexamethasone for relapsed multiple myeloma. N. Engl. J. Med..

[144] Cacan E., Ozmen Z.C. (2020). Regulation of fas in response to bortezomib and epirubicin in colorectal cancer cells. J. Chemother..

[145] Okazuka K., Ishida T. (2018). Proteasome inhibitors for multiple myelomaJap. J. Clin. Oncol..

[146] Kuhn D.J., Chen Q., Voorhees P.M., Strader J.S., Shenk K.D., Sun C.M., Demo S.D., Bennett M.K., van Leeuwen F.W., Chanan-Khan A.A. (2007). Potent activity of carfilzomib, a novel, irreversible inhibitor of the ubiquitin-proteasome pathway, against preclinical models of multiple myeloma. Blood.

[147] Jakubowiak A.J., Dytfeld D., Griffith K.A., Lebovic D., Vesole D.H., Jagannath S., Al-Zoubi A., Anderson T., Nordgren B., Detweiler-Short K. (2012). A phase 1/2 study of carfilzomib in combination with lenalidomide and low-dose dexamethasone as a frontline treatment for multiple myeloma. Blood.

[148] Richardson P.G., Zweegman S., O’Donnell E.K., Laubach J.P., Raje N., Voorhees P., Ferrari R.H., Skacel T., Kumar S.K., Lonial S. (2018). Ixazomib for the treatment of multiple myeloma. Expert Opin. Pharmacother..

[149] Moreau P., Masszi T., Grzasko N., Bahlis N.J., Hansson M., Pour L., Sandhu I., Ganly P., Baker B.W., Jackson S.R. (2016). Oral ixazomib, lenalidomide, and dexamethasone for multiple myeloma. N. Engl. J. Med..

[150] Petroski M.D. (2008). The ubiquitin system, disease, and drug discovery. BMC Biochem..

[151] Cheon K.W., Baek K.H. (2006). Hausp as a therapeutic target for hematopoietic tumors (review). Int. J. Oncol..

[152] Issaeva N., Bozko P., Enge M., Protopopova M., Verhoef L.G., Masucci M., Pramanik A., Selivanova G. (2004). Small molecule rita binds to p53, blocks p53-hdm-2 interaction and activates p53 function in tumors. Nature Med..

[153] Vassilev L.T., Vu B.T., Graves B., Carvajal D., Podlaski F., Filipovic Z., Kong N., Kammlott U., Lukacs C., Klein C. (2004). In vivo activation of the p53 pathway by small-molecule antagonists of mdm2. Science.

[154] Tovar C., Rosinski J., Filipovic Z., Higgins B., Kolinsky K., Hilton H., Zhao X., Vu B.T., Qing W., Packman K. (2006). Small-molecule mdm2 antagonists reveal aberrant p53 signaling in cancer: Implications for therapy. Proc. Natl. Acad. Sci. USA.

[155] Stuhmer T., Chatterjee M., Hildebrandt M., Herrmann P., Gollasch H., Gerecke C., Theurich S., Cigliano L., Manz R.A., Daniel P.T. (2005). Nongenotoxic activation of the p53 pathway as a therapeutic strategy for multiple myeloma. Blood.

[156] Colland F., Formstecher E., Jacq X., Reverdy C., Planquette C., Conrath S., Trouplin V., Bianchi J., Aushev V.N., Camonis J. (2009). Small-molecule inhibitor of usp7/hausp ubiquitin protease stabilizes and activates p53 in cells. Mol. Cancer Ther..

[157] Chitta K., Paulus A., Akhtar S., Blake M.K., Caulfield T.R., Novak A.J., Ansell S.M., Advani P., Ailawadhi S., Sher T. (2015). Targeted inhibition of the deubiquitinating enzymes, usp14 and uchl5, induces proteotoxic stress and apoptosis in waldenstrom macroglobulinaemia tumour cells. Br. J. Haematol..

[158] Vogel R.I., Pulver T., Heilmann W., Mooneyham A., Mullany S., Zhao X., Shahi M., Richter J., Klein M., Chen L. (2016). Usp14 is a predictor of recurrence in endometrial cancer and a molecular target for endometrial cancer treatment. Oncotarget.

[159] Liu Y., Xu X., Lin P., He Y., Zhang Y., Cao B., Zhang Z., Sethi G., Liu J., Zhou X. (2019). Inhibition of the deubiquitinase usp9x induces pre-b cell homeobox 1 (pbx1) degradation and thereby stimulates prostate cancer cell apoptosis. J. Biol. Chem..

[160] Byun S., Lee S.Y., Lee J., Jeong C.H., Farrand L., Lim S., Reddy K., Kim J.Y., Lee M.H., Lee H.J. (2013). Usp8 is a novel target for overcoming gefitinib resistance in lung cancer. Clin. Cancer Res..

[161] Liu Y., Lashuel H.A., Choi S., Xing X., Case A., Ni J., Yeh L.A., Cuny G.D., Stein R.L., Lansbury P.T. (2003). Discovery of inhibitors that elucidate the role of uch-l1 activity in the h1299 lung cancer cell line. Chem. Biol..

[162] Tian Z., D’Arcy P., Wang X., Ray A., Tai Y.T., Hu Y., Carrasco R.D., Richardson P., Linder S., Chauhan D. (2014). A novel small molecule inhibitor of deubiquitylating enzyme usp14 and uchl5 induces apoptosis in multiple myeloma and overcomes bortezomib resistance. Blood.

[163] Mistry H., Hsieh G., Buhrlage S.J., Huang M., Park E., Cuny G.D., Galinsky I., Stone R.M., Gray N.S., D’Andrea A.D. (2013). Small-molecule inhibitors of usp1 target id1 degradation in leukemic cells. Mol. Cancer Ther..

[164] Liang Q., Dexheimer T.S., Zhang P., Rosenthal A.S., Villamil M.A., You C., Zhang Q., Chen J., Ott C.A., Sun H. (2014). A selective usp1-uaf1 inhibitor links deubiquitination to DNA damage responses. Nat. Chem. Biol..

[165] Chen J., Dexheimer T.S., Ai Y., Liang Q., Villamil M.A., Inglese J., Maloney D.J., Jadhav A., Simeonov A., Zhuang Z. (2011). Selective and cell-active inhibitors of the usp1/uaf1 deubiquitinase complex reverse cisplatin resistance in non-small cell lung cancer cells. Chem. Biol..

[166] Davis M.I., Pragani R., Fox J.T., Shen M., Parmar K., Gaudiano E.F., Liu L., Tanega C., McGee L., Hall M.D. (2016). Small molecule inhibition of the ubiquitin-specific protease usp2 accelerates cyclin d1 degradation and leads to cell cycle arrest in colorectal cancer and mantle cell lymphoma models. J. Biol. Chem..

[167] Issaenko O.A., Amerik A.Y. (2012). Chalcone-based small-molecule inhibitors attenuate malignant phenotype via targeting deubiquitinating enzymes. Cell Cycle.

[168] Okada K., Ye Y.Q., Taniguchi K., Yoshida A., Akiyama T., Yoshioka Y., Onose J., Koshino H., Takahashi S., Yajima A. (2013). Vialinin a is a ubiquitin-specific peptidase inhibitor. Bioorg. Med. Chem. Lett..

[169] Chauhan D., Tian Z., Nicholson B., Kumar K.G., Zhou B., Carrasco R., McDermott J.L., Leach C.A., Fulcinniti M., Kodrasov M.P. (2012). A small molecule inhibitor of ubiquitin-specific protease-7 induces apoptosis in multiple myeloma cells and overcomes bortezomib resistance. Cancer Cell.

[170] Weinstock J., Wu J., Cao P., Kingsbury W.D., McDermott J.L., Kodrasov M.P., McKelvey D.M., Suresh Kumar K.G., Goldenberg S.J., Mattern M.R. (2012). Selective dual inhibitors of the cancer-related deubiquitylating proteases usp7 and usp47. ACS Med. Chem. Lett..

[171] Pal A., Young M.A., Donato N.J. (2014). Emerging potential of therapeutic targeting of ubiquitin-specific proteases in the treatment of cancer. Cancer Res..

[172] Kapuria V., Peterson L.F., Fang D., Bornmann W.G., Talpaz M., Donato N.J. (2010). Deubiquitinase inhibition by small-molecule wp1130 triggers aggresome formation and tumor cell apoptosis. Cancer Res..

[173] Zhou B., Zuo Y., Li B., Wang H., Liu H., Wang X., Qiu X., Hu Y., Wen S., Du J. (2013). Deubiquitinase inhibition of 19s regulatory particles by 4-arylidene curcumin analog ac17 causes nf-kappab inhibition and p53 reactivation in human lung cancer cells. Mol. Cancer Ther..

[174] D’Arcy P., Linder S. (2012). Proteasome deubiquitinases as novel targets for cancer therapy. Int. J. Biochem. Cell Biol..

[175] Wang X., D’Arcy P., Caulfield T.R., Paulus A., Chitta K., Mohanty C., Gullbo J., Chanan-Khan A., Linder S. (2015). Synthesis and evaluation of derivatives of the proteasome deubiquitinase inhibitor b-ap15. Chem. Biol. Drug Des..

